# Karyotype of Polyommatus (Agrodiaetus) eriwanensis Forster, 1960 and taxonomic position of P. (A.) interjectus de Lesse, 1960 (Lepidoptera, Lycaenidae)

**DOI:** 10.3897/CompCytogen.v13i4.46897

**Published:** 2019-11-12

**Authors:** Vladimir A. Lukhtanov, Alexander V. Dantchenko

**Affiliations:** 1 Department of Karyosystematics, Zoological Institute of the Russian Academy of Sciences, Universitetskaya nab. 1, St. Petersburg 199034, Russia; 2 Department of Entomology, St. Petersburg State University, Universitetskaya nab 7/9, St. Petersburg 199034, Russia; 3 Faculty of Chemistry, Lomonosov Moscow State University, GSP-1, Leninskiye Gory 1/11, Moscow119991, Russia

**Keywords:** Armenia, Turkey, biodiversity, chromosome, DNA barcoding, meiosis, karyosystematics

## Abstract

The karyotype of Polyommatus (Agrodiaetus) eriwanensis Forster, 1960 from the type locality (“Eriwan” [Yerevan, Armenia]) and other localities in Armenia was investigated. The number of chromosomal elements (bivalents+ multivalents) observed in male meiosis I was found to vary from 29 to 34. In individuals with n = 34, all observed elements were represented by bivalents. In other specimens, heterozygosity for different number of chromosomal fusions resulted in multivalent formation at MI stage and consequently in a lower number of recognizable chromosomal elements. We show that all karyotype peculiarities of P. (A.) interjectus de Lesse, 1960 (n = 29–32) from Turkey are similar to those in *A. eriwanensis*. The butterflies of these taxa have allopatric distribution and can be considered as conspecific.

## Introduction

Polyommatus (Agrodiaetus) eriwanensis Forster, 1960 is a little-known taxon that was originally described as a subspecies *Agrodiaetus ripartii eriwanensis* Forster, 1960 from “Eriwan” [Yerevan, Armenia]. According to molecular data, this taxon belongs to the *P. dolus* (Hübner, 1823) clade of the subgenus Agrodiaetus Hübner, 1822 (Kandul et al. 2007; Vishnevskaya et al. 2016). Based on the morphological characters, this species belongs to a group of so-called anomalous blue butterflies [known also as the ‘brown complex’ of the subgenus Agrodiaetus and as the *Polyommatus admetus* (Esper, 1783) species complex] (Vishnevskaya et al. 2016). This group is composed of multiple species in which both male and female butterflies have similar brown coloration on the upperside of the wings (Lukhtanov et al. 2003).

The anomalous blue butterflies represent a real stumbling block in *Agrodiaetus* taxonomy (Lukhtanov et al. 2003; Przybyłowicz et al. 2014). Despite morphological similarity, the species of the ‘brown complex’ demonstrate a high level of differentiation with respect to chromosome number and karyotype structure (de Lesse 1960; Lukhtanov et al. 2015; Vershinina and Lukhtanov 2017; Vishnevskaya et al. 2018). Therefore, as in other *Agrodiaetus* clades (Lukhtanov 2015; Vershinina et al. 2015; Lukhtanov and Dantchenko 2017; Lukhtanov and Shapoval 2017), cytogenetic characters represent the most important tool for solving taxonomic problems.

The karyotype of P. (A.) eriwanensis was studied first by Lukhtanov and Dantchenko (2002) who reported a high level of chromosome number variation in this taxon with haploid chromosome number (n) ranging from n = 28 to n = 35. However, the cytogenetic mechanisms explaining this variation were not studied previously. Here we provide the first detailed analysis of the karyotype of P. (A.) eriwanensis and its comparison with the karyotypes of closely related species.

## Material and methods

Fresh (not worn) adult males were used to investigate the karyotypes. After capturing a butterfly in the field, it was placed into a glassine envelope for 1–2 hours to keep it alive until we are ready to process it. Then the butterfly was killed by pinching it firmly on the thorax. Immediately after killing it, the testes were removed from the abdomen and placed into a small 0.5 ml vial with a freshly prepared Carnoy fixative (ethanol and glacial acetic acid, 3:1). Testes were stored in the fixative for 1–12 months at +4 °C. Then the gonads were stained in 2% acetic orcein for 30–60 days at +18–20 °C and analyzed as previously described (Lukhtanov and Dantchenko 2002; Lukhtanov 2017). Haploid chromosome numbers (n) were counted in metaphase I (MI), metaphase II (MII) and anaphase I (AI) cells. The following abbreviation is used in this paper: *ca* is circa, approximately determined chromosome number; n is haploid chromosome number.

### Results and discussion

From numerous specimens collected in Armenia in 1996, 1997, 2001 and 2007 only 14 males from four different populations showed metaphase cells which were acceptable for karyotype analysis.

**Figures 1–7.**
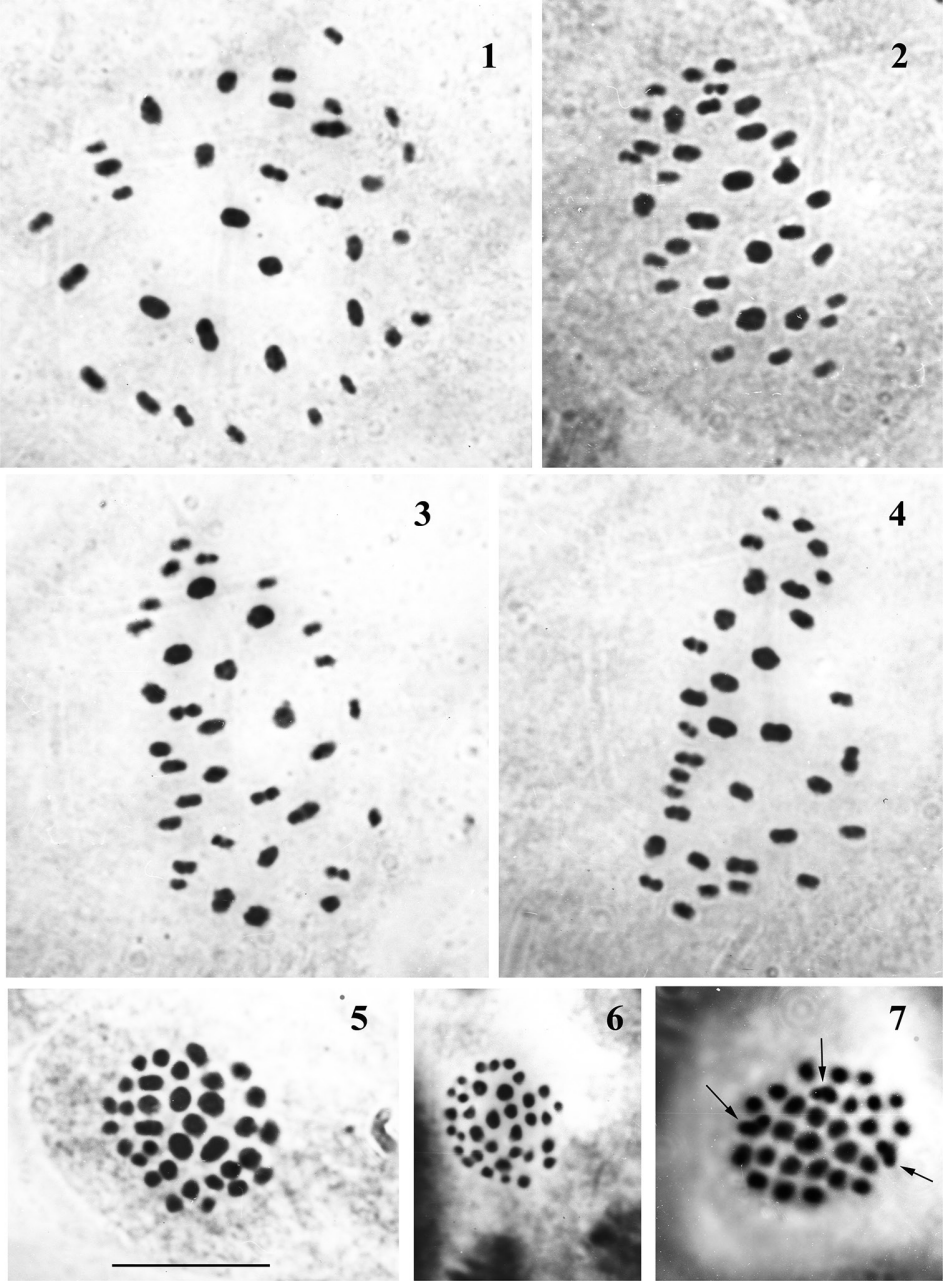
Karyotype in male meiosis of Polyommatus (Agrodiaetus) eriwanensis**1–4** metaphase I of meiosis, squash preparation, n = 34 **5** metaphase I of meiosis, intact (not squashed) preparation, n = 34 **6** metaphase II of meiosis, intact (not squashed) preparation, n = 34 **7** metaphase I of meiosis, intact (not squashed) preparation, n = 29, multivalents are shown by arrows. Scale bar: 10 μm in all figures.

The variable number of distinct chromosome elements was found in MI, MII and AI cells of the studied specimens. In six individuals, 34 chromosome elements were counted (Table 1, Figs 1–6); all observed elements were distinct bivalents in MI and AI cells. Thus, the haploid chromosome number was n = 34 and the diploid number may be calculated as 2n = 68 for these six individuals. In the chromosome set with n = 34, the bivalents were oval or dumb-bell shaped. They were differentiated with respect to their size: the area of the first bivalent was 4–5 times as large as that of the last bivalent (Figs 1–4). In two cells the number of chromosome elements was determined as n = *ca*34 with an approximation due of imperfect spreading of bivalents.

**Table 1.** The number of chromosome elements (bivalents+ multivalents) observed in MI cells of the studied specimens of P. (A.) eriwanensis.

**Code number of the specimen**	**Locality and date of collecting**	**The number of chromosome elements (bivalents+ multivalents) observed**	**Number of cells checked**
KL-1996-34-1	Armenia, Aragaz Mt, ca30 km NW Yerevan, 14-17.07.1997	*ca*32	3MI
KL-1997-6-1	Armenia, Garny, ca15 km E Yerevan, 02.07.1997	*ca*34	7MI
KL-1997-6-4	Armenia, Garny, ca15 km E Yerevan, 02.07.1997	n = *ca*31	1MI
KL-1997-6-7	Armenia, Garny, ca15 km E Yerevan, 02.07.1997	n = 34	1MI
KL-1997-6-8	Armenia, Garny, ca15 km E Yerevan, 02.07.1997	n = *ca*34	1MI
KL-1997-6-9	Armenia, Garny, ca15 km E Yerevan, 02.07.1997	33	8MI
KL-1997-7	Armenia, Garny, ca15 km E Yerevan, 02.07.1997	29	4MI
KL-1997-76-1	Armenia, Aiodzorsky Range, Gnyshik, ca90 km SE Yerevan, 22.07.1997	n = 34	21MI and AI; 2 MII
AD2001-Nr4	Armenia, Geghadir	n = *ca*30	4MI
AD2001-008	Armenia, Aiodzorsky Range, Gnyshik, ca90 km SE Yerevan, 22.07.1997	n = 34	5MI
001A07	Armenia, Aiodzorsky Range, Gnyshik, ca90 km SE Yerevan, loc. 2, 07.2007	n = 34	4MI
002A07	Armenia, Aiodzorsky Range, Gnyshik, ca90 km SE Yerevan, 07.2007	n = 32	5MI
004A07	Armenia, Aiodzorsky Range, Gnyshik, ca90 km SE Yerevan, loc. 2, 07.2007	n = 32	3MI
004A09	Armenia, Aiodzorsky Range, Gnyshik, ca90 km SE Yerevan, 07.2007	n = *ca*32	2MI

If the number of chromosome elements was lower than 34 (29, *ca*30, *ca*31, *ca*32 or 33), from 1 to 6 V-shaped large elements were observed in MI cells. We treat these V-shaped elements as multivalents. For example, in the specimens KL-1997-6-9, 8 cells showed clearly 32 bivalents + 1 multivalent (possibly a trivalent). A very interesting karyotype was found in the specimen KL-1997-7. In this specimen in all studied MI plates we could find 29 chromosome elements including oval or dumb-bell shaped bivalents and three V-shaped multivalents (Fig. 7). The karyotype contained no exceptionally big or small bivalents or multivalents. We suppose that studied cells were heterozygous for multiple chromosome fusions. Small chromosomes of the standard n = 34 karyotype were probably involved in these rearrangements and were observed as parts of the multivalents. Thus, it is evident that the difference in the number of visible chromatin bodies in MI plates does not reflect the real variation of diploid number. Heterozygosity for chromosome fusion(s) may result in multivalent formation at MI stage and consequently in change of number of recognizable chromosome units.

Thus, the number of chromosome elements (bivalents+multivalents) observed in MI was found to vary from 29 to 34. Similar chromosome numbers were found in the following species of the *P. dolus* clade: n = 29-32 in P. (A.) interjectus (de Lesse, 1960) (de Lesse 1960), n = 38 in P. (A.) timfristos Lukhtanov, Vishnevskaya & Shapoval, 2016 (Vishnevskaya et al. 2016), n = 39 in P. (A.) humedasae (Toso & Balletto, 1976) (Vila et al. 2010), n = 41-42 in P. (A.) orphicus Kolev, 2005 (Kolev 2005), n = 40–42 in P. (A.) dantchenkoi (Lukhtanov & Wiemers, 2003) (Lukhtanov et al. 2003) and n = 43 in P. (A.) rjabovianus masul Lukhtanov, Dantchenko, Vishnevskaya & Saifitdinova, 2015 (Lukhtanov et al. 2015).

**Figure 8.**
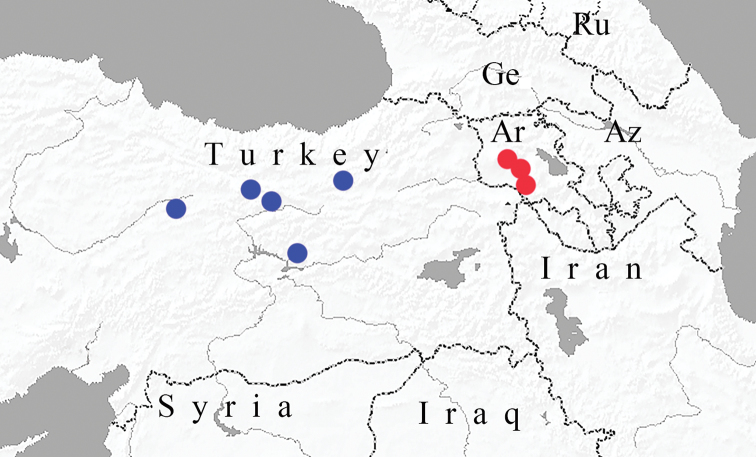
Geographic distribution of P. (A.) eriwanensis eriwanensis (red circles) and P. (A.) eriwanensis interjectus (blue circles) based on information from Hesselbarth et al. (1995) and Lukhtanov et al. (2003). Ar is Armenia, Az is Azerbaijan, Ge is Georgia, Ru is Russia.

We calculated barcoding gaps between these taxa using published *COI* sequences (Lukhtanov et al. 2003; Wiemers 2003; Kandul et al. 2004; Vila et al. 2010; Vishnevskaya et al. 2016) deposited in GenBank (Table 2). As one can see, the species pair *P. eriwanensis* – *P. interjectus* is characterized by a minimum *COI* barcoding gap which is much lower than ‘standard’ DNA-barcode species threshold (3%) (Hebert et al. 2003; Lukhtanov et al. 2016). This pair is also characterized by overlapping numbers of chromosomes. In the MI and MII karyotype of P. (A.) interjectusde Lesse (1960) found from 29 up to 32 chromosome entities with variable number of small and large elements. On the pictures in his paper one can see distinct V-shaped large elements, which are probably multivalents. The chromosome elements were strongly differentiated in P. (A.) interjectus with respect to their size: the area of the largest elements was 4–5 times as large as that of the smallest ones (de Lesse 1960: figs 7 and 8). All these peculiarities are similar to those in P. (A.) eriwanensis. These taxa have allopatric distributions (Fig. 8). Therefore, the taxon studied by de Lesse (1960) can be treated as a subspecies P. (A.) eriwanesis interjecus rather than a distinct species.

**Table 2.** The *COI* barcoding gap (i.e. uncorrected p-distance between the two closest sequences found in the studied pair) and chromosome number distance (difference between the two closest chromosome numbers found in the studied pair).

**Pair of taxa**	**Minimal COI p-distance (barcoding gap)**, %	**Chromosome number distance**
*P. eriwanensis* – *P. interjectus*	0.8	0
*P. eriwanensis* – *P. timfristos*	2.0	4
*P. eriwanensis* – *P. humedasae*	2.5	5
*P. eriwanensis* – *P. orphicus*	2.3	7
*P. eriwanensis* – *P. dantchenkoi*	0.8	6
*P. eriwanensis* – *P. rjabovianus masul*	2.6	9

*Polyommatus eriwanensis* is found in southern Armenia (nominotypical subspecies) and in Ezincan, Erzurum, Sivas and Tunceli Provinces in Turkey (*P. eriwanensis interjectus*) (Fig. 8). The species is also reported for southern Georgia and western Azerbaijan (Eckweiler and Bozano 2016), as well as for north-western Iran (Tshikolovets et al. 2014). However, the indications for Georgia, Azerbaijan and Iran have not been confirmed by chromosomal or molecular data, and we consider them doubtful.
